# State of the Art of Remote Sensing Data: Gradient Pattern in Pseudocolor Composite Images

**DOI:** 10.3390/jimaging12010023

**Published:** 2026-01-04

**Authors:** Alexey Terekhov, Ravil I. Mukhamediev, Igor Savin

**Affiliations:** 1 Institute of Information and Computing Technology, Almaty 050010, Kazakhstan; aterekhov1@yandex.ru; 2 Institute of Automation and Information Technologies, Satbayev University (KazNRTU), Almaty 050013, Kazakhstan; ravil.muhamedyev@gmail.com; 3 V.V. Dokuchaev Soil Science Institute, Moscow 119017, Russia

**Keywords:** remote sensing data, pseudocolor composite image, image texture analysis, gradient pattern, local binary pattern, nominal scale, rank scale, neighborhood matrix

## Abstract

The thematic processing of pseudocolor composite images, especially those created from remote sensing data, is of considerable interest. The set of spectral classes comprising such images is typically described by a nominal scale, meaning the absence of any predetermined relationships between the classes. However, in many cases, images of this type may contain elements of a regular spatial order, one variant of which is a gradient structure. Gradient structures are characterized by a certain regular spatial ordering of spectral classes. Recognizing gradient patterns in the structure of pseudocolor composite images opens up new possibilities for deeper thematic images processing. This article describes an algorithm for analyzing the spatial structure of a pseudocolor composite image to identify gradient patterns. In this process, the initial nominal scale of spectral classes is transformed into a rank scale of the gradient legend. The algorithm is based on the analysis of Moore neighborhoods for each image pixel. This creates an array of the prevalence of all types of local binary patterns (the pixel’s nearest neighbors). All possible variants of the spectral class rank scale composition are then considered. The rank scale variant that describes the largest proportion of image pixels within its gradient order is used as a final result. The user can independently define the criteria for the significance of the gradient order in the analyzed image, focusing either on the overall statistics of the proportion of pixels consistent with the spatial structure of the selected gradient or on the statistics of a selected key image region. The proposed algorithm is illustrated using analysis of test examples.

## 1. Introduction

Some of the most information-rich images are the results of processing of satellite data obtained by scanning the Earth’s underlying surface. In this case, the color formula for each pixel embodies the result of a complex transformation of remote sensing spectral data. The standard approach to processing such information includes various procedures. These include primary geometric and radiometric correction procedures necessary to ensure data comparability [1]. For example, the calculation of physical reflectivity of the underlying surface in selected spectral bands [2], as well as the transformation procedures themselves, of calibrated spectral data, carried out within various types of thematic processing.

The long-term operation of natural resource satellite systems for monitoring the Earth’s surface has allowed the accumulation of large series of remote sensing data. Currently, information describing periods of several decades is available, for example, the EOS MODIS archives (since 1999) [3] or LANDSAT (since 1987) [4]. Thus, it has become possible to obtain robust, long-term estimates of the spectral characteristics of the Earth’s surface and parameters associated with them, including characteristics of long-term change trends [5]. The availability of long-term estimates, which can be components of the analyzed images, has complicated thematic processing due to the ability to solve more complex problems, thereby increasing the need for the development of new image processing algorithms.

The list of mathematical methods for analyzing the spectral characteristics of the underlying surface, applied within the framework of thematic analysis, is quite extensive. It is possible to only note certain areas of this diversity. For example, the calculation of spectral indices, which are understood as the arithmetic of individual spectral bands, which allows us to identify and quantitatively describe many types of characteristic objects on the underlying surface. For example: vegetation indices [6]; water indices [7], soil indices [8]; mineralogical indices [9]. More complex mathematical approaches are used to assess changes in the underlying surface, which may be associated with the presence of long-term trends [10] or seasonal cycles [11]. In all these cases, additional clustering procedures for spectral data sets can be applied for deeper analysis [12]. The development of computing power in recent years has facilitated significant progress in intelligent analysis methods, such as machine learning [13] and AI [14].

The goal of thematic processing of spectral remote sensing data is always to reduce the dimensionality of the analyzed phase space by identifying internal patterns. Ideally, the dimensionality of such a phase space should be reduced to one as a result of processing, which best quantitatively describes the target thematic feature. The resulting image represents a final map of the spatial distribution of the thematic feature.

In practically important cases, the nature of the thematic feature is complex and multifaceted and often cannot be simply described. These may include, for example, the degree of desertification [15] or the stages of land degradation [16], land cover forecasts [17], including agricultural land status [18], and many others. In this case, multivariate models based on wide sets of external parameters can be used to determine the mathematical relationships between the original spectral characteristics and the thematic feature. For example, the WOFOST model, used to describe the growth and development of agricultural crops [19]. Such models typically have many input parameters, only some of which represent information associated with remote sensing data. When considered at a regional scale, information on many parameters becomes less accessible and is replaced by expert assessments, which makes the modeling results partly subjective and reduces their accuracy. The new algorithm proposed in this study, which aims to identify gradient patterns within analyzed images, enables empirically expanded thematic interpretation capabilities, which is particularly significant for pseudocolor composite images generated from remote sensing spectral data and their processed products. This new strategy, based on detecting the actual presence of gradient structures in analyzed images and describing them within a rank scale, allows for the identification of additional relationships existing in the phase feature space, thereby enhancing the effectiveness of remote sensing data thematic interpretation.

In fact, the new approach to thematic processing enables the construction of empirical equations for the relationship between the initial spectral parameters used to construct the analyzed image and the recorded textural features of the image, representing gradient structures. It also allows for varying the spectral parameters during image generation to obtain more pronounced gradient structures. This methodology is somewhat similar to spectral indices. However, unlike the fixed ratio of spectral bands, which typically describe physically clear properties of the underlying surface, spectral indices use arbitrary sets of spectral information that can describe complex characteristics of spatially distributed structures.

Similar approaches can be used not only for the analysis of remote sensing data, but also for solving other problems related to automated image analysis, for example, problems of analyzing data obtained using meteorological radars [20,21,22] or problems of image analysis in medicine [23].

## 2. Materials and Methods

### 2.1. Principles of Recalculation of the Results of Clustering of Remote Sensing Data into Ranking Scales

Remote sensing data, including satellite images, require preliminary processing before its practical use, for which there is a set of methods and techniques [24]. The depth of remote sensing data processing is associated with the type of scales used to describe the results obtained. The simplest is the nominal scale, which is a simple list of the present independent spectral classes, in which the order of the relative positions of the classes in the scale does not matter. A more complex case arises when there are significant relationships between spectral classes. The description of such situations can be based on rank scales. The presence of a rank order allows sorting and assigning ranks (1st, 2nd, 3rd, etc.) to the available spectral classes and thus obtaining deeper estimates of the structure of the results of thematic processing. The possibility of quantitatively accounting for the differences between spectral classes of different ranks leads to the use of interval scales or coefficient scales. The last two cases are variants of the most widely used types of quantitative scales of spectral classes used in the quantitative processing of remote sensing data [25].

The result of a standard unsupervised classification/clustering procedure for remote sensing data is described by a nominal scale, i.e., a set of individual independent spectral classes. However, in reality, certain types of relationships may still be present in the final results. For example, if the analyzed spectral image of the underlying surface was closely related to territorial properties that can be described within the concept of gradient, the texture of the images obtained through clustering may contain information that allows for further ordering of the spectral classes within the rank order of gradient structures. Identifying the presence of such capabilities represents a new and important task, the solution of which could significantly improve the information content of thematic image analysis based on remote sensing data obtained after unsupervised clustering.

The presence of a gradient order in the clustering results implies the existence of an ordinal scale (K1, K2, K3, …, Kn) for spectral classes applicable to the entire analyzed image, which imposes certain restrictions on the spatial mutual arrangement of pixels of different spectral classes. Any spectral class within the ordinal scale (Km), with the exception of marginal ones (the first and last), should ideally be adjacent to only two classes adjacent to it on the scale, i.e., km-1 and Km+1. This state corresponds to the presence of a gradient structure described by a series consisting of classes K1, K2, K3, …, Kn. For example, in a thermal image of a monotonic gradient temperature field in the temperature range from 0 to +30 °C, with a change step of 1 °C, pixels characterizing +20 °C will border only each other or with pixels displaying a temperature of +19 °C or +21 °C. Gradient textures of any kind present in the analyzed graphic scene will also be organized similarly. However, of course, certain adjacency rules may not be strict, but rather expressed only as statistically expressed preferences.

If the spatial scale of class gradients within an ordinal scale is comparable to the spatial resolution of remote sensing data, or neighborhood rules are not strict, then strict adherence to such a rank order in the immediate neighborhood becomes impossible, and the ranking order may be distorted. In fact, the process of unsupervised clustering of remote sensing data can lead to extremely diverse results, organized in various ways: from truly independent classes forming a texture of random mosaic contrast to ensuring a strictly observed gradient structure.

Therefore, a specialized texture analysis of the results of unsupervised clustering of satellite data, which involves considering the immediate neighborhoods of each pixel, can provide the necessary information for assessing the feasibility of ordering a nominal set of spectral classes within a rank scale. The algorithm developed in this study is designed to evaluate all possible rank ordering options existing in an image of spectral classes and select the best rule that ensures the greatest degree of gradient consistency in pixel arrangement.

### 2.2. Algorithm for Texture Analysis of Spectral Classes on a Regular Lattice

The idea of nearest neighbor analysis for each pixel of the analyzed image is well known and is often used in various image processing methods [26,27,28].

This is also the direction of Cellular Automata, in which dynamic structures are formed by a set of rules for the analysis and transformation of the immediate environment of each cell on regular grids, at each step of discrete time [29]. Also, the literature describes many methods for searching and evaluating various patterns in analyzed images [27,30,31,32]. At the same time, based on the use of similar principles, the solution to a number of specific problems of thematic decoding of remote sensing data is described, for example [33,34,35,36], including the broad field of computer vision [37], or more complex rules for analyzing image texture [38,39,40].

In this study, we describe a new version of an image texture analysis algorithm based on nearest-neighbor statistics for the detection of gradient patterns. Although only the immediate neighborhood of each pixel is analyzed, the results are statistically expressed, describing the presence of preferences for certain types of nearest-neighbor relationships between spectral classes. We also analyze the extent to which these textural features can be described within a rank scale, i.e., consistent with the gradient structure. The gradient structure implies not only the presence of statistically identified types of preferential neighborhoods between spectral classes, but also their consistency and the possibility of ordering them within a rank scale: first, second, third, and so on.

**Figure 1:**
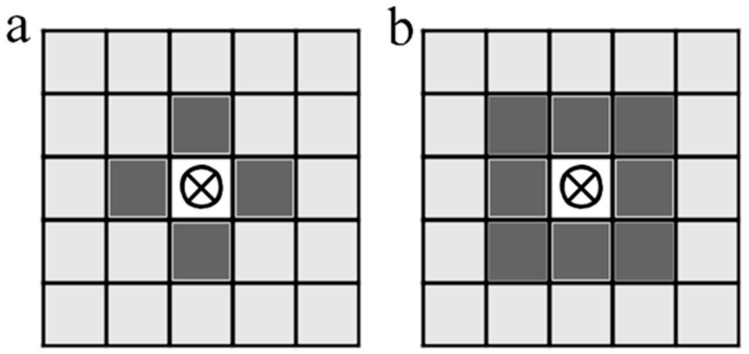
Schemes of accounting for the nearest environment on a square lattice ((**a**) Von Neumann neighborhood; (**b**) Moore neighborhood).

**Figure 2:**
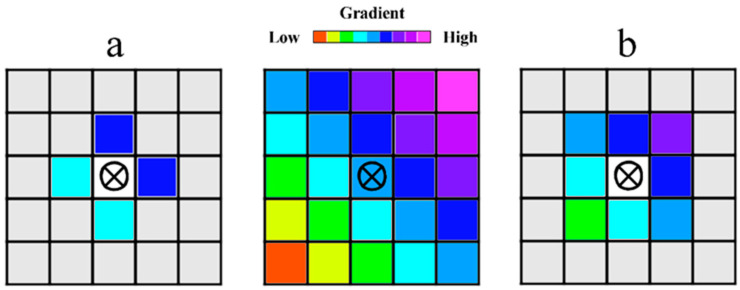
Schemes for identifying cell types in the immediate neighborhood in the case of a simple linear gradient ((**a**) Von Neumann neighborhoods; (**b**) Moore neighborhoods).

The analysis of the relative positions of pixels of different spectral classes in an image is based on the statistics of the immediate neighborhoods of each pixel. There are two possibilities. First, the Von Neumann neighborhood, which is four adjacent cells on a square grid (Figure 1a). Thus, for a cell [i, j] on a two-dimensional square grid, these are the cells with coordinates: [i + 1, j]; [i − 1, j]; [i, j + 1]; [i, j − 1]. Another option is the Moore neighborhood, consisting of 8 cells, which are the von Neumann neighborhood with the addition of four more diagonal cells: [i + 1, j]; [i − 1, j]; [i, j + 1]; [i, j − 1]; [i + 1, j + 1]; [i − 1, j − 1]; [i − 1, j + 1]; [i + 1, j − 1] (Figure 1b) [41]. The Moore neighborhood provides a somewhat larger statistical sample, which makes it preferable, especially in the case of relatively small matrices (Figure 2), but there are no fundamental differences between these options.

**Figure 3:**
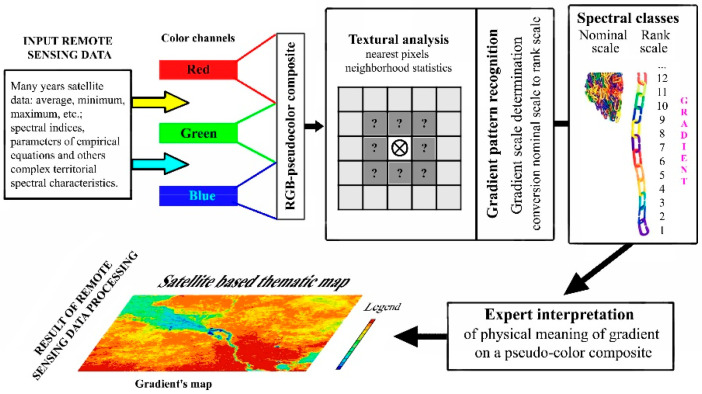
The flowchart of the analysis.

The final result of the statistical neighborhood analysis can be represented as a neighborhood matrix. This matrix contains complete information on the number and types of spectral classes present in the vicinity of Moore, for all spectral classes formed by clustering the satellite data. The proposed algorithm uses an exhaustive search for all possible rank scale variants to find the best one based on the criterion of the largest proportion of image pixels whose spatial arrangement is consistent with the gradient concept. The flowchart of the proposed algorithm is shown in Figure 3.

## 3. Results

### 3.1. Examples of Conversion of Nominal Scale to Ordinal Scale

**Figure 4:**
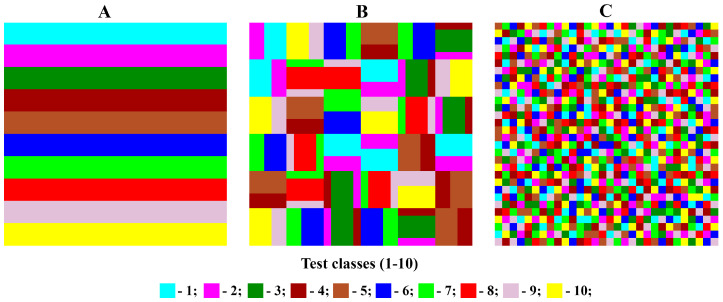
Test matrixes configurations: (**A**) with strictly linear gradient; (**B**) with short-range order; (**C**) with a completely random pixel arrangement.

To demonstrate the operating principles of the proposed new texture analysis algorithm for assessing the presence of a gradient pattern in the morphological structure of an analyzed image, a model case was considered. A regular rectangular two-dimensional lattice of 30 × 30 cells, represented by the corresponding image, was defined. Ten different spectral classes were identified on it. The spatial structure of the image was organized as a simple, uniform, linear gradient along one side, with equal section widths for each class. In this case, the coverage size of each spectral class was 30 × 3 pixels (Figure 4A). Thus, configuration “A” was generated, representing a model image of a simple and strictly linear gradient that can be unambiguously described using an ordinal scale.

To generate configuration “B,” the original 30 × 30 image in configuration “A” was divided into 5 × 5 pixel blocks, for a total of 36 blocks. The new positions of these blocks were determined randomly. Each 5 × 5 pixel block has four possible orientations at its position. Of the four possible positions, one was randomly selected and used to generate configuration matrix “B” (Figure 4B). The new image generated according to these rules retained some short-range order from configuration state “A,” but the long-range order in the gradient structure was completely destroyed.

Configuration “C” had a completely random pixel arrangement. For this purpose, the position of each pixel with a particular class (1–10) in the original matrix “A” was determined randomly. In this case, only the original number of pixels for each of the ten available spectral classes was retained—90 in each (Figure 4C).

**Figure 5:**
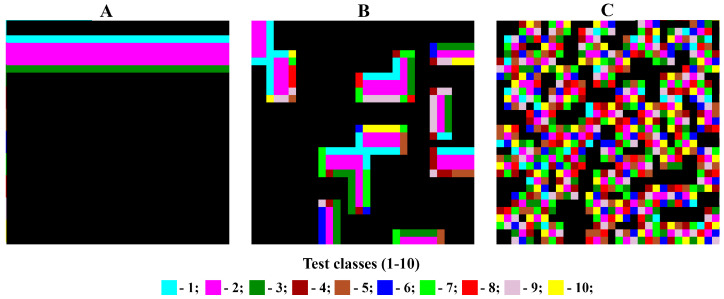
Moore neighborhoods for class 2 (magenta) for different configurations of the test matrix ((**A**) with strictly linear gradient; (**B**) with short-range order; (**C**) with a completely random pixel arrangement).

As an example of selecting and analyzing the composition of the immediate neighborhood, Figure 5 shows cells located in the Moore neighborhood, belonging to spectral class 2 (crimson). The nearest-neighbor matrices for states “A,” “B,” and “C” of the model matrix under consideration are presented in Table 1, Table 2 and Table 3.

The information in the neighborhood matrix reflects the patterns of spatial organization of the analyzed image. Table 1 indicates strict adherence to the gradient structure; most cells have a value of 0, indicating the presence of strict rules prohibiting certain types of neighborhoods. Table 3, in contrast, is characterized by generally high variability and random cell values within a certain spectral range, indicating the absence of any dominant neighborhood types. Table 2 is an intermediate form. While there are no strict rules, there are nevertheless statistically distinguishable preferences for certain types of binary patterns, which can be interpreted as the presence of a noisy gradient.

### 3.2. Satellite Scene Analysis Example

A practical example illustrating the developed algorithm for processing clustering results was considered. The physical nature of the temperature field of the Earth’s surface ensures the automatic formation of gradient structures. Remote sensing data in the thermal channel make it possible to reconstruct temperature fields of the underlying Earth’s surface, which are available in the form of various standard products [42]. Such a temperature field can be considered as a formal source of a graphical scene for its texture analysis in order to identify gradient patterns that should be present a priori. One of the common strategies for thematic processing of a satellite scene involves its clustering using any standard algorithm without training, such as K-means classification [43]. The K-means algorithm determines the membership of spectral classes present in the image to a predetermined number of clusters. The assignment algorithm is based on minimizing the squared sum of the distances between the present spectral classes and the centers of the clusters in the phase space of spectral features. Minimization of the squared sum of the distances is achieved by searching for the optimal positions of a given number of clusters in the feature space through iterative calculations. The clustering results can be analyzed using the proposed algorithm, using nearest neighbor analysis. This will determine the rank order of the spectral classes in the clustering, and based on this, the gradient pattern of the analyzed image can be reconstructed. The degree of identity between the reconstructed gradient field and the actual gradient field present in the original satellite image can serve as a diagnostic indicator of the effectiveness of the proposed approach.

**Figure 6:**
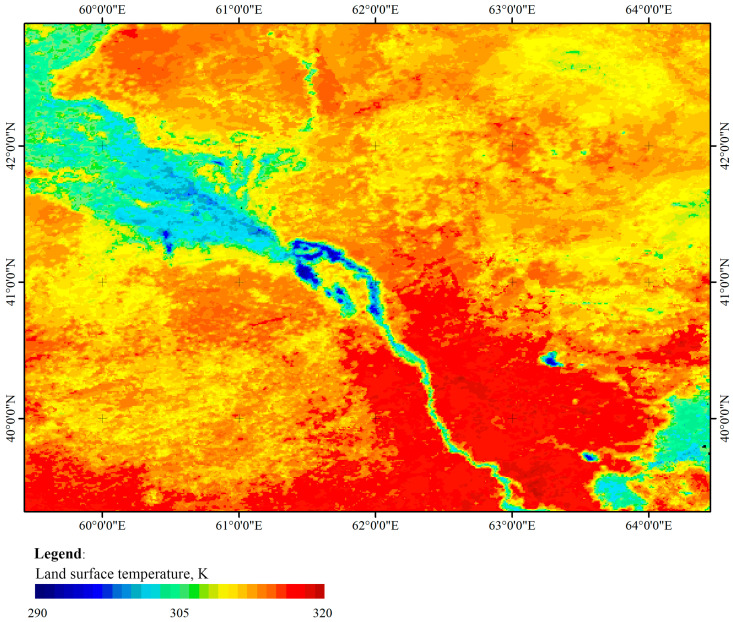
Map of the land surface temperature of southern Kazakhstan, MYD11A2 (V6.1), 24 September 2024.

The MYD11A2 product scene (version 6.1) for 24 September 2024, was used as a practical example (Figure 6). This is a MODIS/Aqua model for determining land surface temperature/emissivity over 8 days on a global L3 grid with an error of 1 km (V061). The satellite imagery was obtained from the Google Earth Engine (GEE) platform [44].

**Figure 7:**
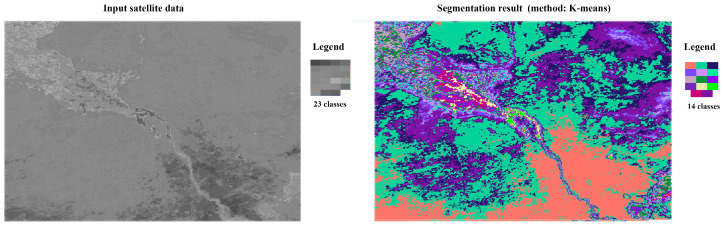
Clustering results of the test satellite scene, MYD11A2, v6.1; 24 September 2024.

Using the Sentinel Application Platform (SNAP) service, the selected temperature map was clustered using the K-means method, generating 14 nominal spectral classes (Figure 7). The number 14 had no deep meaning. It could have been a different number. The goal of clustering was to obtain a pseudocolor composite image with a nominal spectral class scale, but the image actually had a morphological structure closely related to the gradient pattern. The clustering results were subjected to texture analysis with the calculation of a neighborhood matrix (Table 4). The number of pixels of a given spectral class located in the immediate neighborhood of pixels of another class is not in itself indicative. The functional parameter is the proportion of neighboring pixels of a particular selected class relative to all available neighboring pixels of the analyzed spectral class. This recalculation is presented in Table 5. Table 6 presents the ratio of pixels of all spectral classes located in the Moore neighborhood for each class (according to the ordinal numbers of the spectral classes in the most significant rank ordering variant, in terms of the gradient pattern).

Converting from a nominal spectral class scale to a rank scale allows us to divide the pixels of the analyzed matrix into two groups, selecting those that can be assigned to a gradient structure. The gradient structure will include pixels that satisfy the following rule: any pixel of a given spectral class in the gradient structure must be adjacent to only two other classes, either related to adjacent spectral classes within the rank scale or to an identical pixel. The exceptions are the pixels of the first and last spectral classes in the rank scale, which are adjacent to only one class: the first only to the second, and the last only to the penultimate. If the gradient order is strictly observed, all pixels of the analyzed matrix will be associated with the gradient structure. However, in practice, this proportion may be significantly less than 100%.

The total number of ways to transform the nominal spectral class scale into a rank scale is quite large and equals the factorial of the number of spectral classes. For the case under consideration, with 14 spectral classes, this represents just over 87 billion different rank ordering options. The proportion of pixels in the analyzed matrix that can be assigned to the gradient structure with the selected ranking order can serve as a conditional criterion for the significance of this ranking ordering. Thus, by evaluating all possible options, we can determine which ranking option is the most significant—that is, the one in which the proportion of pixels in the analyzed matrix that satisfy the selected gradient structure is highest.

**Figure 8:**
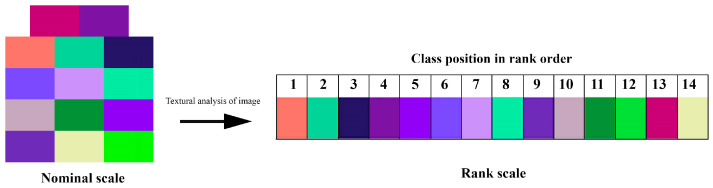
Illustration of the transition from the nominal to the rank scale of spectral classes of the clustering results of the test satellite scene as a result of the analysis of the adjacency matrix (nominal scale—left; rank scale—right).

Analysis of the adjacency matrix of the clustering results for the thermal field test example allowed us to determine the most appropriate rank scale structure (Figure 8).

**Figure 9:**
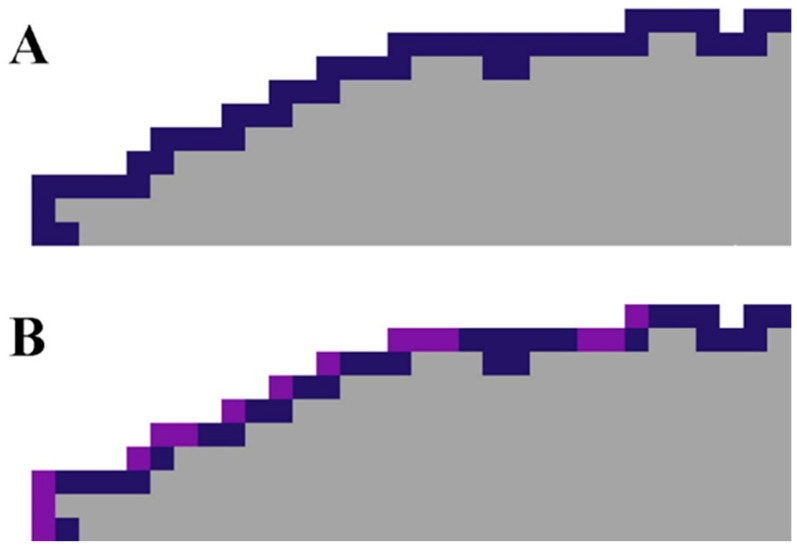
Different variants of the composition of boundary pixels in the Moore neighborhood to the selected spectral class (gray color). (**A**) uniform composition of boundary pixels, in the case of a smooth gradient field on a regular matrix; (**B**) non-uniform composition of boundary pixels (from two different types of spectral classes) in the case of a sharper gradient field on a regular matrix.

The proportion of test matrix pixels satisfying the gradient structure within this ranking option was the maximum possible for the analyzed satellite scene, amounting to 69.504%. The criteria for classifying pixels as belonging to the gradient structure can be expanded by allowing for the possibility of neighboring not two, but four other classes related to the nearest neighbors on the rank scale. This situation corresponds to situations where the gradient field in the satellite scene is too contrasty, and the period of a conventional matrix cannot describe this using a consistent set of spectral classes in accordance with the rank scale. Some classes fall outside the regular order of neighboring classes within the gradient, and configurations of binary patterns appear, adjacent to each other by one position on the rank scale (Figure 9). In this case, the maximum proportion of pixels in the test satellite scene belonging to the gradient structure increased and amounted to 88.358%, that is, the overwhelming majority.

**Figure 10:**
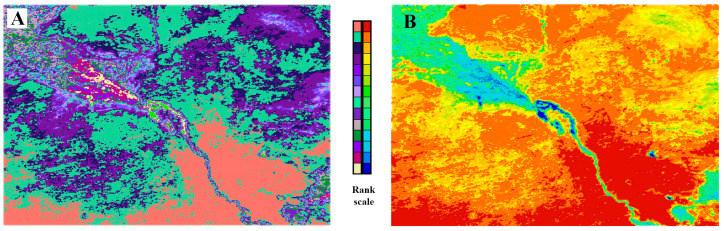
Various graphical representations of the clustering results for 14 spectral classes of the test satellite scene using K-means ((**A**) using the standard graphical palette within the clustering procedure; (**B**) using the temperature field palette for the spectral classes of clustering, ordered within the most significant rank order).

**Figure 11:**
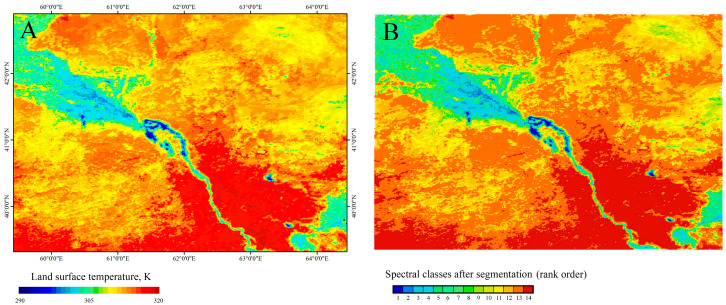
Comparison of the original test satellite scene MYD11A2 (V6.1), 24.09. 2024 and its image, reconstructed after the K-means clustering procedure. ((**A**) original satellite scene LST (30 spectral classes); (**B**) result of K-means clustering (14 spectral classes) after the procedure of ordering the nominal scale of spectral classes into the most significant rank scale).

The spectral class rank order obtained through texture analysis to reconstruct the gradient pattern allows for the color representation of the clustering results to be modified using a color scale typical of quantitative scales, i.e., with a regular and smooth change in the palette’s color parameters, for example, from blue to red. Consequently, it becomes possible to visually compare the graphical structure of the original satellite image of the underlying surface temperature and its image obtained after coarse-grained clustering with the loss of the original spectral class rank order (Figure 10) and its subsequent restoration (Figure 11).

As can be seen in Figure 11, gradient pattern detection using spectral class rank ordering fairly well reconstructed the original morphological gradient structure of the test image. The correlation coefficient between the original image and the result of processing using the proposed algorithm was 0.98.

## 4. Discussion

The above-described algorithm for texture image processing for gradient pattern detection expands the capabilities of spectral information processing by incorporating certain morphological features of the analyzed image. Analysis of the spectral characteristics of pixels in their immediate vicinity provides information on the presence of certain types of preferential neighborhoods (binary patterns), which serves as the basis for diagnosing the presence of gradient patterns and the significance of these morphological features for the entire graphic scene or its individual key areas. The methodology proposed in this study is adapted for the analysis of remote sensing data, including satellite imagery and products based on them, but is not limited to this range of tasks.

Recording and describing gradient patterns in the analyzed graphic scene, such as a pseudocolor composite image, using an appropriate scale can significantly enhance the thematic processing of satellite data. This requires solving two problems. First, recognizing the presence and composition of a gradient. Which spectral classes present in the image, and in what order, form a property gradient. Second, expert determination of the physical meaning of the gradient. The gradient of underlying surface properties is recorded through the identified rank order of spectral classes. This significantly simplifies subsequent thematic interpretation of the obtained clustering results. Expert interpretation of each spectral class becomes unnecessary, only their ensemble within the rank scale describing the gradient structure. It is also desirable to understand the physical meaning of the detected gradient, initially constructing qualitative and, subsequently, possibly quantitative rank estimates.

Geographically distributed objects, including various natural and anthropogenic ones, such as soil or vegetation cover, agricultural land, etc., can be described by various thematic maps using parameters that allow for dependencies expressed as gradients. A new strategy for processing remote sensing data is emerging for such objects. This strategy is based on feedback and optimization. A specific set of initial remote sensing data can provide some description of the gradient structure of the Earth’s underlying surface, serving as the basis for thematic analysis. Consequently, the researcher has the opportunity to vary the initial data to obtain more meaningful gradient patterns. This allows for an empirical search for the most informative remote sensing sources. This innovation is particularly valuable for describing complex processes, including long-term territorial changes, for which the principles of description are unclear and the parameters used can be complex. For example, the degree of desertification, degradation of the underlying surface, or long-term trends in agricultural land characteristics. The morphological features of images containing gradient patterns are quite distinctive. In the case of a linear trend, a key region of the image will have a certain isopotential direction along which spectral classes are quasi-stable. A perpendicular direction will also be present, with a wide variety of spectral class types. The proposed texture processing algorithm provides a metric for the significance of gradient patterns and the composition of the ranking scale for the best description, eliminating the need for purely expert assessments.

To illustrate the practical significance of the above-described idea of using gradient patterns in processing spectral remote sensing data, we can consider the problem of estimating the average long-term secondary salinity of irrigated arable land in southern Kazakhstan. Secondary salinity of irrigated arable land is a dynamic process with varying time scales [45]. There are both intra-seasonal and inter-seasonal dynamics. At the beginning of the growing season, the level of secondary salinity is lower. By the end of the season, it increases. Inter-seasonal dynamics are also significant and are related to weather conditions, particularly the availability of water for irrigation. Dry and wet years differ significantly in the water availability of arid areas in southern Kazakhstan. Taken together, all this creates a complex dynamic picture, making it significantly difficult to describe average long-term conditions. However, it is the average long-term conditions that have the highest practical significance, since they serve as the basis for making management decisions in the field of territorial hydrology and the administration of irrigated agriculture.

Pseudocolor RGB composite images of the southern Kazakhstan region were constructed using Sentinel-2 satellite data, displaying perennial characteristics of vegetation, including irrigated fields. The spectral channels of the RGB composite were formed by analyzing the Sentinel-2 satellite data archive for the period 2018–2022. The Vegetation Soil Salinity Index (VSSI) was used as the RED channel long-term maximum [46].

**Figure 12:**
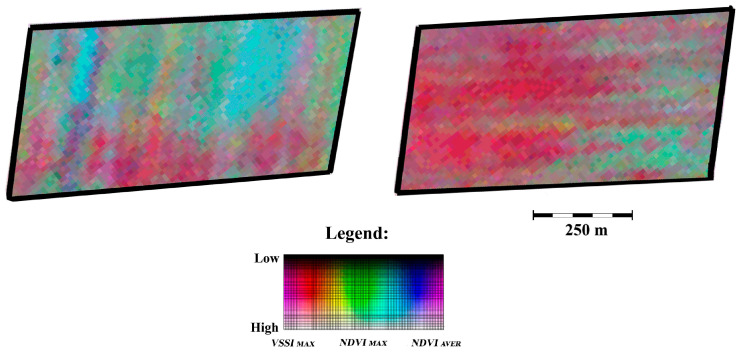
Examples of irrigated fields with a gradient state of soil salinity. Results of processing of Sentinel-2 satellite data. RGB-pseudocolor composite: Red—many years maximum of salinity index (VSSI); Green—many years maximum of vegetation index (NDVI); Blue—many years average of vegetation index (NDVI).

The GREEN channel represented the NDVI vegetation index, in the form of its long-term maximum, and the BLUE channel represented the long-term average NDVI value. In this work, an expert analysis of the RGB composite of the field area with a known gradient structure of the secondary salinity level was carried out, Figure 12. As a result, the expert interpretation restored the rank order of the spectral classes within the framework of their relationship with the level of secondary salinity of arable land, and based on the resulting legend, the original RGB composite was recalculated into a map of the average long-term secondary salinity level [47].

**Figure 13:**
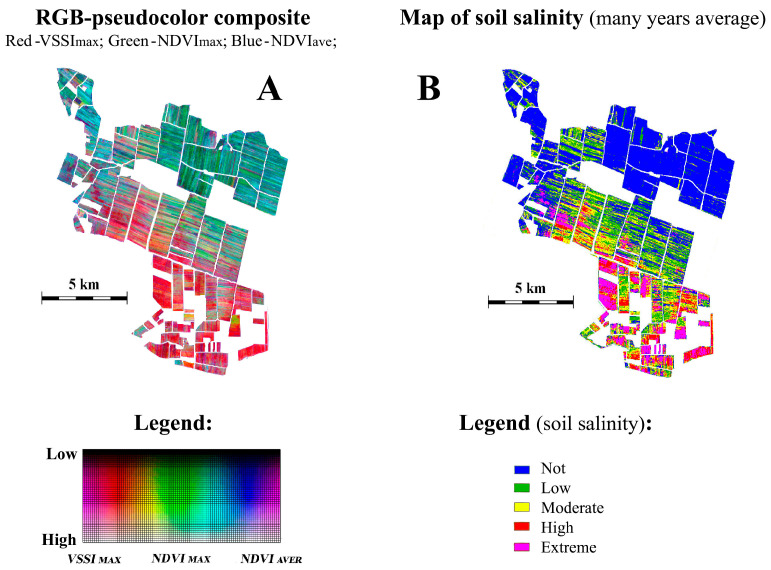
Irrigated arable land of Kyzylkumsky rural district in South Kazakhstan. Results of processing of Sentinel-2 satellite data. (**A**) the result of standard processing procedure; (**B**) the result, taking into account the gradient structure.

The identification and expert analysis of gradient properties in soil salinity (Figure 12) enabled the conversion of the nominal scale of the RGB pseudocolor composite to a scale of average long-term soil salinity (Figure 13). Figure 13 illustrates the importance of morphological analysis based on expert recognition of gradient structures for a more in-depth thematic analysis of the spectral structure of the analyzed graphic scene, for example, in the task of estimating average long-term soil salinity.

Thus, the algorithm described in this paper for converting from a nominal scale of spectral classes to a rank scale expands the capabilities of thematic processing of graphic scenes, including those constructed based on remote sensing or other similar data [48,49], replacing complex and subjective expert analysis. This procedure may be of interest as a new, additional feature in the toolbox of thematic processing of satellite information within the framework of relevant specialized software.

The limitations of using the developed image texture analysis procedure are minimal. Analysis of spectral class types in the immediate vicinity of each pixel in an image can, in principle, be performed for any image. However, the presence of a large number of different spectral classes in the analyzed graphic scene makes the mathematical process of finding the most appropriate gradient scale quite cumbersome. The total number of possible gradient scale combinations, including all spectral classes present in the image, is a factorial of the number of spectral classes. This means that the volume of necessary calculations rapidly increases with the number of classes. Another potential problem is the presence of multiple independent gradients in the image, each described by only a subset of the spectral classes present. In this case, a more advanced analysis algorithm than the one described above will be required, which could become the next step in the development of this field.

## 5. Conclusions

In summary, a new image texture analysis algorithm has been developed based on the statistical analysis of spectral class types in the immediate vicinity of a pixel. The purpose of the analysis was to identify and evaluate the significance of specific morphological image features expressed as gradient patterns. The original spectral classes, initially organized within a nominal scale, are sorted into a rank scale in the gradient legend.

The developed image processing algorithm may be of greatest practical use for thematic analysis of pseudocolor composite images constructed from remote sensing data, including satellite imagery. This opens up new and interesting possibilities for empirically selecting initial remote sensing data that provides better descriptions of territories using gradient patterns.

In the future, this texture analysis algorithm may be further modified to adapt it to specific sets of original data and thematic problems being solved. For example, pseudonearest neighbor analysis is an interesting option. It is also likely that complexly structured pseudocolor images may contain multiple spectrally independent gradient patterns, the analysis of which will enable deeper thematic data processing.

The developed image processing algorithm could form the basis for a separate, additional function in satellite data processing tools within specialized software.

## Figures and Tables

**Table 1:** Neighborhood matrix for the test matrix in configuration “A”.

	**Classes**										
**Classes**		**1**	**2**	**3**	**4**	**5**	**6**	**7**	**8**	**9**	**10**
	**1**	-	30	0	0	0	0	0	0	0	0
	**2**	30	-	30	0	0	0	0	0	0	0
	**3**	0	30	-	30	0	0	0	0	0	0
	**4**	0	0	30	-	30	0	0	0	0	0
	**5**	0	0	0	30	-	30	0	0	0	0
	**6**	0	0	0	0	30	-	30	0	0	0
	**7**	0	0	0	0	0	30	-	30	0	0
	**8**	0	0	0	0	0	0	30	-	30	0
	**9**	0	0	0	0	0	0	0	30	-	30
	**10**	0	0	0	0	0	0	0	0	30	-

**Table 2:** Neighborhood matrix for the test matrix in configuration “B”.

	**Classes**										
**Classes**		**1**	**2**	**3**	**4**	**5**	**6**	**7**	**8**	**9**	**10**
	**1**	-	40	3	11	4	8	8	6	5	9
	**2**	42	-	34	12	9	9	18	6	19	10
	**3**	5	34	-	30	5	8	8	3	3	3
	**4**	11	12	32	-	35	14	13	11	20	12
	**5**	4	8	3	33	-	5	15	6	15	2
	**6**	5	8	6	11	6	-	34	3	22	5
	**7**	8	17	6	11	16	35	-	36	21	11
	**8**	8	8	3	15	6	3	32	-	30	0
	**9**	6	19	3	23	10	19	23	32	-	32
	**10**	9	11	3	14	2	5	12	0	34	-

**Table 3:** Neighborhood matrix for the test matrix in configuration “C”.

	**Classes**										
**Classes**		**1**	**2**	**3**	**4**	**5**	**6**	**7**	**8**	**9**	**10**
	**1**	-	30	58	66	74	59	70	70	69	67
	**2**	30	-	53	64	70	64	72	60	69	62
	**3**	54	47	-	58	65	68	62	63	59	64
	**4**	58	63	57	-	37	62	68	67	57	59
	**5**	65	69	59	37	-	45	54	55	54	55
	**6**	61	61	62	58	47	-	45	50	52	58
	**7**	64	70	59	63	50	47	-	54	60	58
	**8**	63	60	65	65	53	54	54	-	59	55
	**9**	63	66	61	55	58	53	61	60	-	49
	**10**	63	61	61	56	54	59	55	57	49	-

**Table 4:** Neighborhood matrix of clustering results for the test satellite scene. Clustering formed 14 spectral classes. The table shows the number of pixels in the Moore neighborhood for each class (random order of assigning spectral class ordinal numbers).

		**Class ID**													
		**1**	**2**	**3**	**4**	**5**	**6**	**7**	**8**	**9**	**10**	**11**	**12**	**13**	**14**
**Class ID**	**1**	-	19,651	0	265	0	17	30,143	145	465	0	75	866	1	3
	**2**	19,415	-	72	1688	0	139	9332	732	183	0	401	5095	10	5
	**3**	0	68	-	214	2409	3637	10	476	4	20	1281	127	560	237
	**4**	284	2307	235	-	83	510	212	2473	18	1	1296	3603	35	22
	**5**	0	0	2637	83	-	929	5	196	4	60	424	35	1718	637
	**6**	19	156	3508	494	921	-	10	1197	6	28	3368	198	269	176
	**7**	23,448	8170	16	157	4	12	-	87	10,637	0	38	398	2	3
	**8**	128	911	480	2506	187	1225	103	-	17	9	2934	1327	66	42
	**9**	408	158	1	15	3	6	11,074	16	-	17	3	22	2	1
	**10**	0	0	35	1	48	26	0	9	10	-	11	0	73	425
	**11**	62	369	1272	1248	406	3684	44	2656	5	10	-	593	138	115
	**12**	1011	7687	112	3121	37	204	538	1319	31	0	576	-	13	9
	**13**	1	9	639	39	1837	281	3	68	3	101	135	13	-	1190
	**14**	2	3	265	18	541	158	3	36	1	365	108	9	1190	-

**Table 5:** Neighborhood matrix of clustering results for the test satellite scene. The table shows the proportions of pixels of all spectral classes located in the Moore neighborhood for each class (random order of assignment of spectral class ordinal numbers).

		**Class ID**													
		**1**	**2**	**3**	**4**	**5**	**6**	**7**	**8**	**9**	**10**	**11**	**12**	**13**	**14**
**Class ID**	**1**	-	0.3806	0	0.0051	0	0.0003	0.5838	0.0028	0.0090	0	0.0015	0.0168	0	0
	**2**	0.5237	-	0.0019	0.0455	0	0.0037	0.2517	0.0197	0.0049	0	0.0108	0.1374	0.0003	0.0001
	**3**	0	0.0075	-	0.0237	0.2664	0.2664	0.0011	0.0526	0.0004	0.0022	0.1417	0.0140	0.0619	0.0262
	**4**	0.0256	0.2082	0.0212	-	0.0075	0.0460	0.0191	0.2232	0.0016	0	0.1170	0.3252	0.0032	0.0020
	**5**	0	0	0.3919	0.0123	-	0.1381	0.0007	0.0291	0.0006	0.0089	0.0630	0.0052	0.2554	0.0947
	**6**	0.0018	0.0151	0.3389	0.0477	0.0890	-	0.0010	0.1156	0.0006	0.0027	0.3254	0.0191	0.0260	0.0170
	**7**	0.5457	0.1901	0.0004	0.0037	0	0.0003	-	0.0020	0.2475	0	0.0009	0.0093	0	0
	**8**	0.0113	0.0917	0.0483	0.2522	0.0188	0.1233	0.0104	-	0.0017	0.0009	0.2953	0.1336	0.0066	0.0042
	**9**	0.0348	0.0135	0.0001	0.0013	0.0003	0.0005	0.9444	0.0014	-	0.0015	0.0003	0.0019	0.0002	0.0001
	**10**	0	0	0.0549	0.0016	0.0752	0.0408	0	0.0141	0.0157	-	0.0172	0	0.1144	0.6661
	**11**	0.0058	0.0348	0.1200	0.1177	0.0383	0.3475	0.0042	0.2505	0.0005	0.0009	-	0.0559	0.0130	0.0108
	**12**	0.0690	0.5244	0.0076	0.2129	0.0025	0.0139	0.0367	0.0900	0.0021	0	0.0393	-	0.0009	0.0006
	**13**	0.0002	0.0021	0.1480	0.0090	0.4253	0.0651	0.0007	0.0157	0.0007	0.0234	0.0313	0.0030	-	0.2755
	**14**	0.0007	0.0011	0.0982	0.0067	0.2004	0.0585	0.0011	0.0133	0.0004	0.1352	0.0400	0.0033	0.4409	-

**Table 6:** Neighborhood matrix of the clustering results of the test satellite scene within the selected rank scale of spectral classes. The table shows the proportions of pixels of all spectral classes located in the Moore neighborhood for each class (assignment of ordinal numbers of spectral classes within the most significant rank order).

		**Class ID**													
		**1**	**2**	**3**	**4**	**5**	**6**	**7**	**8**	**9**	**10**	**11**	**12**	**13**	**14**
**Class ID**	**1**		0.9444	0.0348	0.0135	0.0019	0.0013	0.0014	0.0003	0.0005	0.0001	0.0003	0.0002	0.0001	0.0015
	**2**	0.2475	-	0.5457	0.1901	0.0093	0.0037	0.0020	0.0009	0.0003	0.0004	0.0001	0	0.0001	0
	**3**	0.0090	0.5838	-	0.3806	0.0168	0.0051	0.0028	0.0015	0.0003	0	0	0	0.0001	0
	**4**	0.0049	0.2517	0.5237	-	0.1374	0.0455	0.0197	0.0108	0.0037	0.0019	0	0.0003	0.0001	0
	**5**	0.0021	0.0367	0.0690	0.5244	-	0.2129	0.0900	0.0393	0.0139	0.0076	0.0025	0.0009	0.0006	0
	**6**	0.0016	0.0191	0.0256	0.2082	0.3252	-	0.2232	0.1170	0.0460	0.0212	0.0075	0.0032	0.0020	0.0001
	**7**	0.0017	0.0103	0.0129	0.0915	0.1333	0.2517	-	0.2947	0.1231	0.0482	0.0189	0.0066	0.0042	0.0009
	**8**	0.0005	0.0042	0.0058	0.0348	0.0559	0.1177	0.2505	-	0.3475	0.1200	0.0383	0.0130	0.0108	0.0009
	**9**	0.0006	0.0010	0.0018	0.0151	0.0191	0.0477	0.1157	0.3254	-	0.3389	0.0890	0.0260	0.0170	0.0027
	**10**	0.0004	0.0011	0	0.0075	0.0140	0.0237	0.0526	0.1417	0.4022	-	0.2664	0.0619	0.0262	0.0022
	**11**	0.0006	0.0007	0	0	0.0052	0.0123	0.0291	0.0630	0.1381	0.3919	-	0.2554	0.0947	0.0089
	**12**	0.0007	0.0007	0.0002	0.0021	0.0030	0.0090	0.0157	0.0313	0.0651	0.1480	0.4253	-	0.2755	0.0234
	**13**	0.0004	0.0011	0.0007	0.0011	0.0033	0.0067	0.0133	0.0400	0.0585	0.0982	0.2004	0.4409	-	0.1352
	**14**	0.0157	0	0	0	0	0.0016	0.0141	0.0172	0.0408	0.0549	0.0752	0.1144	0.6661	-

## Data Availability

No new data were created or analyzed in this study. Data sharing is not applicable to this article.

## Abbreviations

The following abbreviations are used in this manuscript:

SNAP	Sentinel Application Platform
